# Autism-Like Behavior in the Offspring of *CYP11A1*-Overexpressing Pregnant Rats

**DOI:** 10.3389/fnins.2021.774439

**Published:** 2021-12-23

**Authors:** Tianying Pan, Chuan Jiang, Juan Cheng, Jiang Xie, Xinghui Liu, Wenming Xu, Guolin He

**Affiliations:** ^1^Key Laboratory of Birth Defects and Related Diseases of Women and Children of Ministry of Education, Department of Gynecology and Obstetrics, West China Second University Hospital, Sichuan University, Chengdu, China; ^2^Reproductive Endocrinology and Regulation Laboratory, West China Second University Hospital, Sichuan University, Chengdu, China; ^3^Third People’s Hospital of Chengdu, Chengdu, China

**Keywords:** *CYP11A1* gene, autism, microglial immunity, GABAA receptor, testosterone

## Abstract

Autism spectrum disorders (ASD) represent a complex group of neurodevelopmental disorders that are characterized by impaired social behavior and communication as well as repetitive behavior and restricted interests. Prenatal exposure to high levels of testosterone and preeclampsia are thought to be risk factors of ASD. We had previously reported that overexpression of the mitochondrial cholesterol side-chain cleavage enzyme (CYP11A1) could lead to both preeclampsia-like symptoms and increased testosterone levels in pregnant rats. In this study, we investigated the association between high CYP11A1 levels in pregnant rats and autism-like behavior in their offspring. Timed-pregnant Sprague-Dawley (SD) rats were injected with *CYP11A1* gene-carrying adenoviruses on gestational day 8.5, and their offspring were then compared with those from timed-pregnant control SD rats. Compared with their control counterparts, the offspring of the *CYP11A1*-ovexpressing dams displayed more symptoms of anxiety and spent less time in social interactions and more time in self-grooming and rearing, all indicators of autism-like behavior. Sequencing of the transcriptome in primary microglia from the offspring of *CYP11A1*-overexpressing dams revealed that immune pathways were highly activated, and the gamma-aminobutyric acid type A (GABAA) receptor genes were among the top differentially expressed genes. Using primary microglia cultures generated from neonatal rats, tumor necrosis factor-alpha expression was found to be elevated in the cells transfected with *CYP11A1*-carrying adenoviruses. Additionally, the offspring of *CYP11A1*-overexpressing dams displayed dysregulated GABAA receptor expression. Taken together, these results suggest that abnormal *CYP11A1* gene expression in pregnant rats could lead to microglial immune activation and dysregulated GABAA receptor expression in their offspring and thereby anxiety and autism-related behavior. Our study suggests that the pathways regulated by CYP11A1 could be promising preventative and therapeutic targets for ASD.

## Introduction

Autism spectrum disorders (ASD) are a complex group of heterogeneous neurodevelopmental disorders characterized by communication and social interaction deficits and restricted, repetitive, and stereotypical behaviors (Lai et al., 2014; Lord et al., 2018). The incidence of autism has risen significantly in the last two decades, with a 2012 systematic review estimating the global prevalence to be approximately 1% (Elsabbagh et al., 2012). Although prenatal exposure to high levels of testosterone and preeclampsia are thought to be risk factors of ASD (Baron-Cohen et al., 2011), the exact mechanism underlying the pathogenesis of this condition remains unclear. Using animal models, researchers have shown that the treatment of dams with testosterone may lead to autism-like behavior in their offspring (Xu et al., 2015). Furthermore, a prospective study on human males showed that elevated fetal steroidogenic activity *in utero* could result in an increased incidence of later development of autism (Baron-Cohen et al., 2015). This critical role of testosterone is attributed, at least in part, to dysregulation of the microglia in newborn offspring. Researchers have also shown that there were significant differences in microglial gene expression between males and females, which could partially explain the higher incidence of ASD in males (Mccarthy and Wright, 2017).

The human *CYP11A1* gene encodes a member of the cytochrome P450 superfamily of enzymes that catalyzes the conversion of cholesterol to pregnenolone, the first and rate-limiting step in the synthesis of the steroid hormones. Pregnenolone is a precursor for progesterone and its downstream hormones. We had previously shown that placental *CYP11A1* expression was increased in patients with preeclampsia and that the overexpression of this enzyme could lead to the apoptosis of placental trophoblasts (He et al., 2013). In another study, we showed that *CYP11A1* overexpression induced preeclampsia-like symptoms in rats (Pan et al., 2017). Moreover, the compromised migratory and invasive abilities of trophoblasts were associated with testosterone and androgen receptor-mediated pathways (Pan et al., 2017). Recent genetic association studies have shown that single nucleotide polymorphisms (SNPs) of *CYP11A1* were related to the pathogenesis of autism (Shen et al., 2014; Deng et al., 2016). On the basis of these findings, we hypothesized that *CYP11A1* overexpression in the pregnant mother may induce autism-like behavior in her offspring. In this study, we found that abnormal *CYP11A1* gene expression in pregnant rats could induce anxiety and autism-related behaviors in their offspring through the regulation of microglial immune activation and the dysregulation of gamma-aminobutyric acid type A (GABAA) receptor (GABAR) expression. Our study suggests that the pathways regulated by CYP11A1 could be promising preventative and therapeutic targets for ASD.

## Materials and Methods

### Animals and Treatment

Sprague–Dawley (SD) female (nulliparous; 240–260 g) and male rats (280–310 g), which were obtained from Chengdu Dossy Experimental Animal Inc. (Chengdu, China), were housed under a 12/12 h light/dark cycle with free access to food and water. After the females had been housed overnight with fertile males at a 1:1 ratio, their vaginal plug was observed in the morning. The presence of a vaginal plug was considered a confirmation of pregnancy and designated gestational day (GD) 0.5. The parturition day was considered to be postnatal day 1. All experimental procedures were carried out according to the Ethical Principles in Animal Research and were approved by the Ethics Committee on the Use of Animals of Sichuan University. The timed-pregnant rats were then randomly divided into two groups of 10 dams each. On GD 8.5, the animals in one group (hereinafter the “test” group/dams) received an injection of *CYP11A1* gene-carrying adenoviruses (AD-CYP11A1, 15879-1, GeneChem), whereas those in the other group (hereinafter the “control” group/dams) received an injection of the control adenovirus vehicle (Product No:ADCON177, GeneChemM). The adenovirus we used was CMV-MCS-3FLAG-SV40-EGFP. Moreover, Cyp11a1 full length cDNA (NM_000781) was inserted into the adenovirus construct. Empty plasmid was used as a control. Both groups received injections containing 1 × 10^9^ plaque-forming units (PFU) of the green fluorescent protein (GFP)-tagged vector into their tail veins. Pups were born through normal vaginal delivery on GD 20–22. After weaning, the pups were housed under a 12/12 h light/dark cycle with free access to food and water. At the ages of 6–8 weeks, they were subjected to the combined open field maze and social behavioral test, elevated plus maze (EPM) test, and water maze test. Then, the offspring from both groups were sacrificed, and fresh microglia were harvested for RNA sequencing as well as hippocampus and cortex tissues for western blot analysis. Additionally, fresh microglia were harvested from newborn pups of untreated SD rats and infected with AD-CYP11A1 (described below).

### Elevated Plus Maze Test

The elevated plus maze (EPM) arena, which was elevated 55 cm above the floor, consisted of four black floors and two open arms (50 × 10 cm) without walls and two closed arms (50 × 10 × 30 cm) enclosed by 30-cm-high walls extending from a central platform (10 × 10 cm). Offspring (6–8 weeks old) from the test and control dams were placed individually onto the junction of the open and closed arms. Only male rats were used to negate the influence of the estrous cycle on EPM behavior. The number of entries and time spent on the open and closed arms were recorded over a 10 min period using EPM software (BW-DEP207, Shanghai Biowill Co., Ltd.). After each trial, the arms and center area were cleaned with 70% alcohol and left to dry before reuse.

### Open Field Maze Test and Social Behavioral Analysis

The open field maze consisted of a square box of 50 × 50 cm width and 30 cm height. Paired stranger offspring of the same gender from the test or control dams were simultaneously placed in the center of the open field. The number and duration of contacts were recorded with a video recorder for 10 min and analyzed using a social behavioral analysis system (BW-Social LAB, Shanghai Biowill Co., Ltd.). Grooming and rearing were coded by two trained observers who were blinded to the experimental conditions. Grooming was defined as self-licking of the body and/or self-rubbing of the face or fur with the front paws. Rearing was defined as the number of times the animal stood on its hind paws in the field.

### Water Maze Test

To assess the spatial learning set acquisition of the rats, a black water maze of 120 cm in diameter with a 10 cm circular platform was used. For 6 consecutive days, the offspring from the test and control dams underwent one trial per day, which lasted for 2 min with a 30 s inter-training interval. On the first day of training, the platform was exposed one inch above the surface, with direction signs on the walls of the testing room. During the second and fifth days, the platform was placed in the same position but one inch below the surface, with the same direction signs on the testing room walls. Rats were placed at four different starting positions, from east to north, respectively, facing the tank wall. Any rat unable to find the platform was guided to it and left on the platform during the 30 s interval. On the sixth day, the direction signs were withdrawn, and the rats were placed randomly at one of the four positions. The latency and path length were measured using a tracking program (BW-MWM101, Shanghai Biowill Co., Ltd).

### Cell Culture

The primary microglia culture, used as an *in vitro* cell model, was generated using a previously described method (Tamashiro et al., 2012) with modifications. In brief, brain tissues were harvested from newborn pups of three untreated SD rats, which had been anesthetized using the hypothermia method. The tissues were thoroughly cut and then washed three times with Dulbecco’s modified Eagle’s medium. Next, 0.25% trypsin and DNase were added to the cut samples, and the mixtures were then incubated in a 37°C water bath for 20 min. After the incubation, an equal amount of DMEM medium was added to stop the digestion process. The samples were then transferred to DMEM medium and blown gently until there were no obvious tissue masses remaining. The digested tissue samples were transferred to new centrifuge tubes for centrifugation. Thereafter, the supernatant was discarded, the cell pellet was resuspended in medium, and the cell density was quantified. An appropriate amount of cells was then seeded in 75 cm^2^ flasks.

The cultures were maintained until astrocytes covered the bottom of the culture flask (∼9–16 days). The flasks were then placed on a shaker and agitated at 150 rpm at 37°C to allow the microglia to fall off the surface of the astrocytes. After 1 h of shaking, the medium containing the floating microglia was collected and centrifuged, and the cell pellet was resuspended in complete medium and used to inoculate fresh cultures. After 1 h of culture, the medium was changed to remove non-adherent oligodendrocytes, and then, complete medium was added for continued culturing.

After 24 h of culture, the CD11b (bs-1014R-AF488, Bioss) immunofluorescence staining method was used to identify the microglial cells (Zhang et al., 2015). The cells were then treated with 1 × 10^8^ PFU of either AD-CYP11A1 or ADCON177 for 48 h.

For the culturing of GABAergic neurons, N2A cells were obtained from Prof. Li Hedong (West China Second University Hospital, Sichuan University) and cultured using a previously described method (Gao et al., 2016). N2A cells were divided into the following four groups: groups A and B were infected with 1 × 10^8^ PFU of AD-CYP11A1 and control, respectively, for 48 h; group C was treated with 1 μm pregnenolone (IP0360, Solarbio) for 48 h; and group D was left untreated as the control group. Three wells were used per group for data analysis.

### Preparation and Sequencing of mRNA Library

Fresh microglia were harvested from offspring (aged 6–8 weeks) of the test and control dams and quantified, and the microglia were prepared as reported previously with minor modifications (Tamashiro et al., 2012). Place the freshly perfused adult brain in serum free medium into a the 100-mm dish and then mince brain as finely as possible using the 15T scalpel blade. Transfer the minced brain to 50-mL tube containing 10 mL of dissociation medium, Gently rock (or invert the tube every 5 min) cell suspension in tissue culture incubator for 20 min. The cells were isolated with Percoll gradients based on the reference. Total RNA was then extracted from at least 10^6^ fresh cells and dissolved in RNase-free water for the synthesis of complementary DNA (cDNA) using the SMART-Seq™ v4 Ultra™ Low Input RNA Kit for Sequencing (Clontech). After purifying the double-stranded cDNAs using AMPure XP beads, the sequences were amplified using the long-distance polymerase chain reaction (PCR), and their final concentration was quantified using a Qubit fluorometer. An Agilent 2100 bioanalyzer was used to measure the size of the cDNAs, and a HiSeq system was used to sequence the samples. The sequencing data from this study have been submitted to Gene Expression Omnibus under accession no. GSE185805.

### Real Time Polymerase Chain Reaction

Total RNA was extracted by using 1 mL Trizol reagent. Absorbances at 260 and 280 nm were measured to estimate the amount of RNA extracted. The extracted RNA was stored at –80°C. Real time PCR (qPCR) was determined using a Dynamo Flash SYBR Green qPCR kit (Thermo Fisher Scientific) according to the manufacturer’s instructions. qPCR was performed on an ABI 7500 sequence detection system (Life Technologies).

### Western Blot Analysis

N2A cells and hippocampus and cortex tissues were lysed using RIPA buffer (Sigma-Aldrich), and the total protein concentrations were determined using the Pierce BCA Protein Assay Kit. Equal amounts of protein (80 μg) were separated using 10% sodium dodecyl sulfate-polyacrylamide gel electrophoresis and then transferred to a polyvinylidene difluoride membrane for incubation (4°C, overnight) with rabbit monoclonal primary antibodies against the following proteins: gamma-aminobutyric acid type A receptor subunit alpha 1 (GABRA1; 1:2,000 dilution, 12410-1-AP, Proteintech), gamma-aminobutyric acid receptor subunit delta (GABRD; 1:1,000, 15623-1-AP, Proteintech), CYP11A1 (1:5,000, 67264-1-Ig, Proteintech), cAMP-response element binding protein (CREB; 1:1,000, ab32515, Abcam), phosphorylated-CREB (P-CREB; 1:2,000, ab32096, Abcam), and glyceraldehyde 3-phosphate dehydrogenase (GAPDH; 1:10,000, 200306-7E4, Zen-Bio). Then, after washing the membrane with Tween-containing Tris-buffered saline, it was incubated for 1 h with horseradish peroxidase-conjugated secondary antibodies at ambient temperature, following which the bands were visualized using the enhanced chemiluminescence assay. GAPDH was used as the loading control. The optical densities of the bands were quantitatively analyzed using ImageJ software.

### Hormone and TNF-α Measurement Using Enzyme-Linked Immunosorbent Assay and Mass Spectrometry

Steroid levels were determined using enzyme-linked immunosorbent assays (ELISAs). The testosterone levels were determined using a testosterone assay kit (KGE010, R&D Systems) according to the manufacturer’s protocol. Each sample was measured in triplicates. The blood pregnenolone levels in the animals were determined using isotope dilution high-performance liquid chromatography–tandem mass spectrometry as previously described, where the coefficient of variation for the hormone was found to be 9.2% (Zhou et al., 2015).

TNF-α levels were measured by rat TNF-α ELISA Kit (D731168, R&D Systems). Add 100 μl standards and samples in each reaction well and incubated for 90 min at 37°C. Discard the supernatant in the well and dry it. Add 100 μl biotin-conjugated antibody working solution to each reaction well, incubate for 60 min at 37°C, and discard the supernatant and dry it. Add 350 μl washing solution to each reaction well, and incubate with washing solution for 1–2 min. Repeat 4 times, Add 100 μl HRP-conjugated streptavidin working solution to each reaction well, incubate for 30 min at 37°C, add 300 μl washing solution to each reaction well, and leave the washing solution for intervals of 30 s in the well. Repeat this process four times. Add 90 μl substrate reagent (Hiding from light) to each reaction well, the color was developed at 37°C for about 15 min. Then, add 50 μl stop solution to each reaction well, immediately measure the absorbance at a wavelength of 450 nm with a microplate reader (within 5 min).

### Statistical Analysis

Each experiment was conducted in triplicates. Results are expressed as the mean ± standard error of the mean. Differences between two groups were evaluated using the unpaired *t*-test, and data were analyzed using GraphPad Prism 5. Differences were considered statistically significant at a *P*-value of less than 0.05.

## Results

### A Rat Model of *CYP11A1*-Overexpressing Dams Was Successfully Established

**FIGURE 1:**
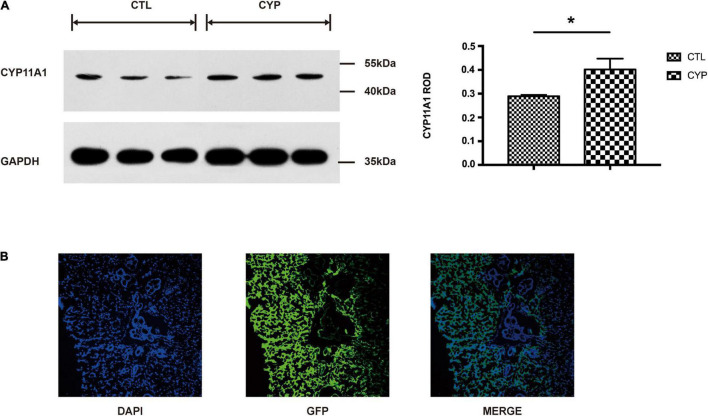
A rat model of *CYP11A1*-overexpressing dams was successfully established. **(A)** Western blot result shows that CYP11A1 adenovirus injection lead to overexpression of CYP11A1 in placenta. The right panel is the statistic result. **(B)** Immunostaining analysis revealed green fluorescent protein (GFP) signaling in the placentas of *CYP11A1*-overexpressing dams. *N* = 3 for each group (*P < 0.05).

Our previous study using a rat model of *CYP11A1* gene overexpression showed that the treated dams developed preeclampsia-like symptoms (Pan et al., 2017). Western blot result showed that the placental expression of CYP11A1 was significantly higher in the AD-CYP11A1-infected rats than in the control rats (Figure 1A). Immunofluorescence staining analysis revealed the presence of green fluorescence of virus particles in the placenta (Figure 1B), confirming the successful establishment of this animal model and that it could be responsible for the observed phenotypes. Results from our previous study had shown that over-expression of CYP11A1 also lead to increased level of pregnenolone in offspring (Pan et al., 2017), indicating that the treatment was responsible for the increased level of the enzyme.

### Offspring From the *CYP11A1*-Overexpressing Dams Showed Increased Anxiety and Reduced Social Behavior

**FIGURE 2:**
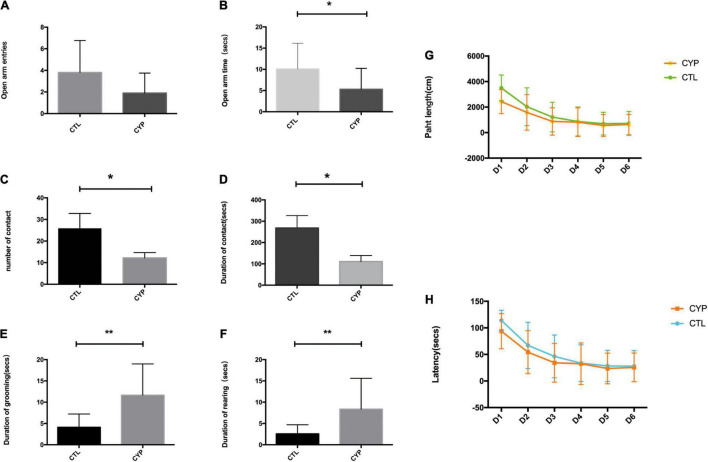
CYP11A1 overexpression in pregnant rats increased anxiety behaviors and reduced social behaviors in their offspring. **(A,B)** Comparisons of entries and times spent in the open arms of the elevated plus maze. Although there was no difference between the offspring of the *CYP11A1*-overexpressing and control groups in the number of entries into the open arms (*N* = 16 for control and *N* = 10 for *CYP11A1*- overexpressing group), the times spent in the open arms were significantly shorter for the offspring of *CYP11A1*-overexpressing dams (*N* = 16 for control, and *N* = 12 for *CYP11A1*- overexpressing group). **(C,D)** The social experiments revealed that the number and duration of contacts were lower for the offspring of *CYP11A1*-overexpressing dams (*N* = 9 for control, and *N* = 18 for *CYP11A1*- overexpressing group). **(E,F)** The incidences of grooming and rearing were significantly increased for the offspring of the *CYP11A1*-overexpressing group (*N* = 20 for control and *N* = 23 for *CYP11A1*- overexpressing group). **(G,H)** There were no significant differences between the offspring of the two groups in terms of the latency and path length results of the water maze test (*N* = 25 for control and *N* = 20 for *CYP11A1*- overexpressing group) (*P < 0.05, **P < 0.01).

The offspring from the two groups of dams were subjected to the EPM test to measure their levels of anxiety. Male offspring were used to negate the influence of the estrous cycle on the test results. The offspring from the test dams displayed elevated anxiety-like behavior, as they made less entries and spent significantly less time in the open arms of the EPM than their control counterparts (Figures 2A,B). In the open field maze, which measures social behavior, the offspring from the test dams had a significantly lower incidence of contact with others and shorter contact durations than the control offspring (Figures 2C,D). By contrast, the rearing incidences were higher and the self-grooming durations significantly longer for the offspring from the test dams (Figures 2E,F). The lower social contact and higher incidences of rearing and self-grooming indicated that the offspring from the test dams had autism-like behavior. However, in the water maze test for assessment of spatial learning and reference memory, there were no significant differences in the latency and path length results between the two groups of offspring (Figures 2G,H).

### Sequencing of the Microglial Transcriptome Revealed Immune Activation in Offspring From the *CYP11A1*-Overexpressing Dams

**FIGURE 3:**
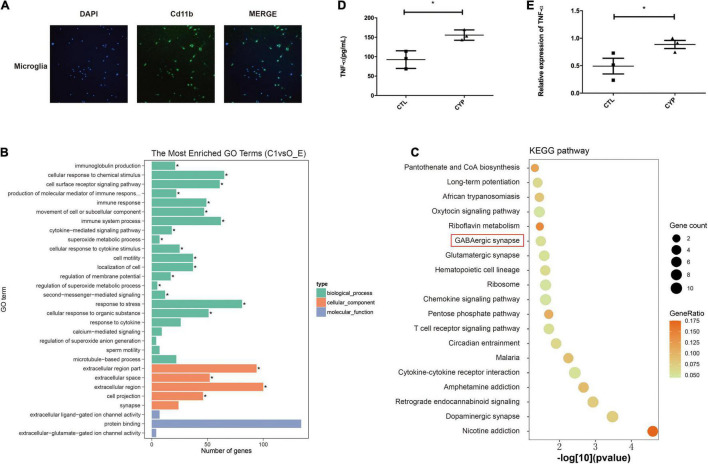
Primary microglial transcriptome and primary culture indicates immune activation in offspring from *CYP11A1*-overexpressing dams. **(A)** Microglial cells from the offspring of AD-CYP11A1-treated pregnant rats were identified by their Cd11b positivity. **(B)** GO analysis revealed the activation of immune responses in primary microglia from the CYP11A1 overexpression group. **(C)** KEGG analysis indicated that GABAergic synapse related genes were enriched from the CYP11A1 overexpression group (over_represented *p* = 0.033). *N* = 3 for each group, and only male rats were used for the transcriptomic analysis. **(D,E)** Expression of the inflammatory tumor necrosis factor-alpha (TNF-α) was increased in the primary microglia in the *CYP11A1* adenovirus infected cells. ELISA revealed TNF-α in the adenovirus infected culture medium **(D)**, and real-time PCR detected TNF-α mRNA in the microglia **(E)** (*P < 0.05).

Microglia, which are major immune cells in the brain, have recently been shown to play a central role in anxiety, autism, and other psychiatric diseases (Kaur et al., 2017; Salter and Stevens, 2017). Therefore, we extracted primary microglia from brain tissue of the offspring from the two groups of dams for transcriptome sequencing to identify the major pathways affected by *CYP11A1* overexpression in these cells. Cd11b staining confirmed the identity of the microglial cells, and the cell purity is 83% based on Cd11b positive rate (Figure 3A). GO pathway analysis of the top differentially expressed genes in the offspring from the test dams revealed that genes were significantly enriched in pathways associated with “production of molecular mediator of immune response” (*P* < 0.01) (Figure 3B). Additionally, genes enriched in “immune response” were significantly dysregulated in these same offspring (*P* < 0.05) (Figure 3B). Furthermore, KEGG analysis indicated that GABAergic synapse pathways were highly activated in cells from the test dam offspring (Figure 3C). Therefore, these results support the hypothesis that microglial cell function may be dysregulated in the offspring from *CYP11A1*-overexpressing dams, which may lead to dysregulation of GABAergic neurons.

### *CYP11A1* Overexpression Activated Inflammation in a Primary Culture Model of Newborn Pup-Derived Microglial Cells

As immune cells, the microglia have a close relationship with the neurons during brain development and are major secretors of cytokines (Kaur et al., 2017; Salter and Stevens, 2017). We extracted microglial cells from newborn pups of untreated rats to test whether their overexpression of CYP11A1 would affect their functions. The cytokine levels were measured in the AD-CYP11A1-infected primary cultures, whereupon both the real-time PCR and ELISA results revealed that the mRNA and protein levels of TNF-α (a major inflammatory factor) were significantly increased relative to the control levels (Figures 3D,E). Taken together, our results indicate that *CYP11A1* overexpression activates inflammation in microglial cells *in vitro*.

### CYP11A1 Regulated GABAergic Receptor Expression in Neurons and in Microglial Cells From Offspring of *CYP11A1*-Overexpressing Dams

**FIGURE 4:**
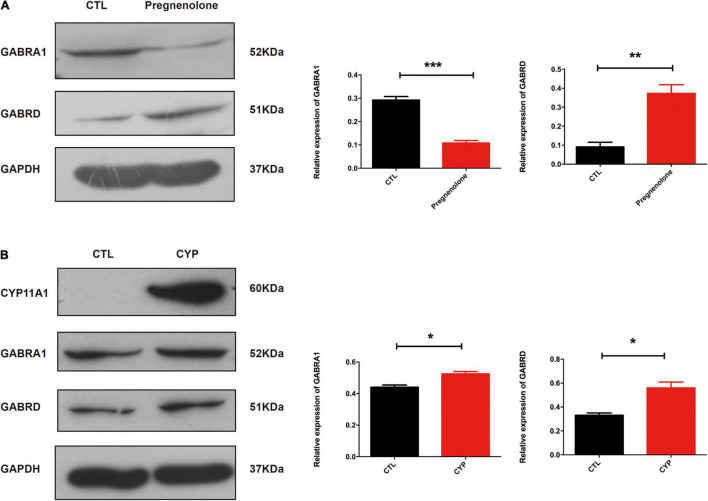
CYP11A1 overexpression lead to altered GABAA receptor expression in primary microglial and neuronal cultures. **(A)** Western blot analysis showed that pregnenolone treatment decreased GABRA1 and increased GABRD expression in N2A neurons. **(B)** GABRA1 and GABRD expression was induced by CYP11A1 overexpression. GAPDH was used as an internal control (*P < 0.05, **P < 0.01, and ***P < 0.001). The experiments were repeated two times.

We had recently reported that progesterone could regulate GABAA activity through its interaction with the P2X_2_ receptor that mediates the calcium influx needed for the acrosome reaction (Xu et al., 2017). In this present study, KEGG pathway analysis of the transcriptome revealed that GABAergic synapses were altered in primary microglial cells from the offspring of the test dams. *De novo* Neurosteroidogenesis was detected in Human Microglia (Germelli et al., 2021). Therefore, we confirmed that *CYP11A1* overexpression in dams affected GABAA receptor subunit expression in their offspring. Next, we determined the effect of pregnenolone (a direct metabolite of the enzymatic action of CYP11A1) on the expression of the GABAA subunit using N2A cells, a cell line which has been shown expressing the major GABAA subunits. The western blotting results indicated that GABRA1 expression was significantly reduced, whereas GABRD expression was significantly increased, in the pregnenolone-treated group (Figure 4A). Interestingly, AD-CYP11A1 transfection significantly increased both the GABRA1 and GABRD expression levels (Figure 4B), indicating that although pregnenolone and CYP11A1 can induce GABRD expression, the discrepancy of GABRA1 expression for adding pregnenolone and transfecting CYP11A1 indicates that although CYP11A1 is the major enzyme responsible for pregnenolone production, possibly other functions or metabolites of CYP11A1 are also involved in GABRA1 regulation in neurons.

**FIGURE 5:**
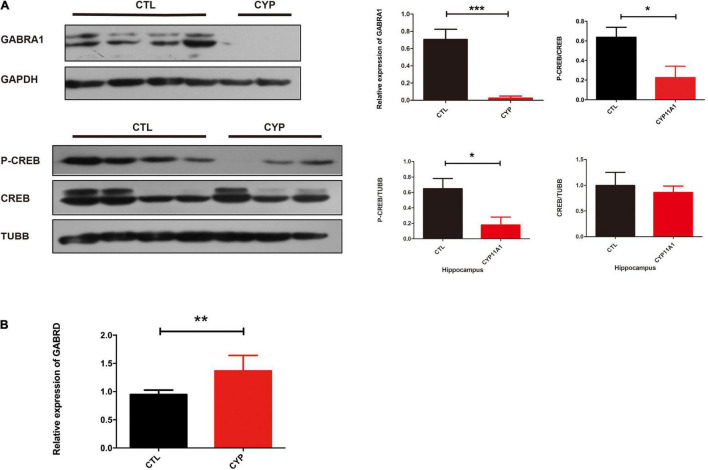
CYP11A1 overexpression *in vivo* led to altered GABAA receptor expression. **(A)** Western blot analysis revealed that CYP11A1 overexpression induced GABRA1 expression and decreased the ratio of P-CREB to CREB in the brain hippocampus. **(B)** GABRD transcript expression was increased by CYP11A1 overexpression (*P < 0.05, **P < 0.01, and ***P < 0.001).

Given that the AD-CYP11A1 infection in pregnant rats led to autism-like neurobehavior in their offspring, and the *in vitro* cell model showed that CYP11A1 treatment altered GABAA subunit expression, we next investigated whether GABAA subunit expression was altered *in vivo*. We have chosen brain regions including hippocampus and cortex, which have been shown relevant for neurobehavior related to autism. Although the cortex lost the neurogenesis in postnatal period, the region has been shown closely related to anxious, social interaction, among others. Western blotting results showed that GABRA1 expression in the hippocampus was significantly reduced (Figure 5A). CREB and p-CREB expression are important markers of neuron activity. CREB is activated by phosphorylation through the activities of extracellular signal-regulated protein kinases (ERK) 1/2, which has been shown to be part of a major hub of signaling pathways in neurogenesis (Pardo et al., 2017). We found that the levels of P-CREB and the ratio of P-CREB to CREB were significantly decreased in the hippocampus from the test group (Figure 5A). Real-time PCR indicated that the GABRD transcripts levels were significantly increased in this test group (Figure 5B). Therefore, the western blotting results indicated dysregulation of GABA receptor expression in the offspring of test dams. This suggested that dysregulated GABA receptor expression may be responsible, at least in part, for the altered neurobehavior of the offspring from the *CYP11A1*-overexpressing dams. Expression of the GABARs in the cortex was also examined and no significant differences were detected (data not shown). Taken together, the study results indicate that dysregulated CYP11A1 expression in pregnant rats could lead to microglial immune activation and dysregulated GABAR expression in the neurons of their offspring, which contribute to their autistic-like traits.

## Discussion

**FIGURE 6:**
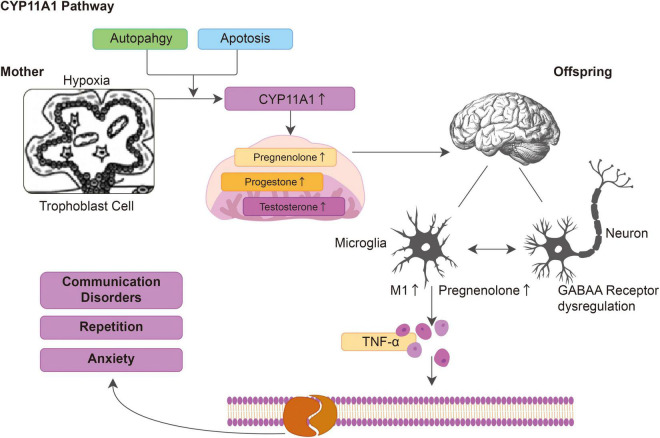
The hypothesized model depicting dysregulated CYP11A1 expression leads to immune activation in microglia and dysregulated GABAA expression in neurons, which contribute to the autistic-like traits in CYP11A1-treated rat offspring.

In this study, we found that abnormal *CYP11A1* gene expression in pregnant rats could induce anxiety and autism-like behavior in their offspring through the regulation of microglial immune activation and dysregulation of GABAR expression in the neurons. *CYP11A1* overexpression in the dams activated immune responses and increased the microglial secretion of inflammatory cytokines, such as TNF-α. *In vitro CYP11A1* transfection led to altered GABAA subunit expression; specifically, GABRA1 and GABRD were dysregulated in the neurons *in vitro* and in an *in vivo* animal model, indicating that inflammation and altered GABAA signaling in neurons were responsible for the neurobehavioral changes in the offspring. The proposed mechanism of how CYP11A1 dysregulation is involved in autism related phenotype is illustrated in Figure 6. Based on the result showed in the current study, we have shown that both genetic variants and environmental insult could induce CYP11A expression, which could induce the production of pregnenolone in the placenta and cortex. The up-regulated pregnenolone and the down-stream metabolites, could activate GABAA receptor subunits, and activate cytokines release. The activated cytokines could affect several aspects of neuron function, including myelin phagocytosis among other functions, and result in autism related symptom. In conclusion, we have shown that during pregnancy, the dysregulated CYP11A1 may closely linked to the etiology of autism in offspring, at least in some subtypes (Figure 6).

CYP11A1 is essential for steroid biosynthesis in the placenta, ovaries, and brain, with the placenta contributing significantly to enzyme activity during pregnancy (Hu et al., 2004). Abnormal CYP11A1 expression may therefore affect steroid levels, which are closely related to various diseases, such as polycystic ovary syndrome (PCOS), breast cancer, and other hormone-related disorders (Chien et al., 2013; Shen et al., 2014; Cesta et al., 2016). In our previous study, we found that *CYP11A1* gene expression was significantly increased in the placenta during severe preeclampsia compared with that in normal pregnancies (He et al., 2013). Considering the recent reports of a close relationship between placental insufficiency and autism (Walker et al., 2015; O’Loughlin et al., 2017), our results from this study confirmed that altered CYP11A1 levels could affect both placental development in the mother and neurobehavior in her offspring. Furthermore, differences in pregnenolone levels could be responsible for the phenotypes observed in the offspring, as the levels of this hormone are altered in children with autism (Fung et al., 2014). Although the rats in our present study were treated with *CYP11A1*-carrying adenoviruses, the real-world effect of CYP11A1 overexpression could have several implications. First, genetic variations in *CYP11A1* could be regulated by SNPs surrounding the regulatory region, as recent genetic association studies have identified an SNP related to the autism phenotype in Chinese children (Shen et al., 2014; Deng et al., 2016). Second, as mentioned above, CYP11A1 levels are often altered in patients with PCOS. A recent report has linked PCOS in mothers to an increased incidence of autism in their children, suggesting that this could be due to the altered CYP11A1 levels in the women (Cesta et al., 2016). Third, a recent study found altered pregnenolone levels in children with autism (Fung et al., 2014), which is similar to the finding in this study that the levels of the hormone were changed in the circulation of the offspring from *CYP11A1*-overexpressing dams. This emphasizes the role of pregnenolone level imbalance in the pathogenesis of autism.

Testosterone is another hormone that has long been known to be related to the etiology of autism, as high androgen levels have been shown to be associated with the pathogenesis of ASD (Pivovarciova et al., 2016; Park et al., 2017). However, the data are conflicting, as other studies failed to find significant associations between changes in postnatal testosterone concentrations in either newborn or children and later autistic traits (Kung et al., 2016a,b). Our study also indicated that testosterone alone cannot induce TNF-α expression in the microglia, indicating that other hormones may be responsible for the link between CYP11A1 expression and autism. Progesterone is another key pregnancy-related hormone (Strauss et al., 2000; Chien et al., 2013), the changes in expression of which can lead to miscarriage and other complications. Whether increased progesterone secretion is involved in the regulation of neurobehavior requires further clarification.

A finding of this study was that *CYP11A1* transfection led to the activation of both microglial immune responses and inflammatory pathways. Given that neurobehavior was changed in the offspring of *CYP11A1*-overexpressing dams, we investigated gene expression in these rats and found that the immune response was activated in the brain microglia. Recent studies have shown that dysregulation of the microglia plays a central role in major psychiatric diseases, such as autism and schizophrenia (Kaur et al., 2017; Turano et al., 2017). One of the most significant of the phenomena in autism is that its incidence is higher in males than in females, and this difference is attributed to the differential microglial responses to sex hormones (Hanamsagar et al., 2017). In fact, our results revealed a more significant phenotype in males than in females when neurobehavior was evaluated (data not shown). In our previous study, we found that the levels of TNF-α and interleukin-6 were significantly increased in children with autism (Xie et al., 2017). In this study, we showed that TNF-α was released from microglia in a primary culture model and that AD-CYP11A1 treatment, but not testosterone treatment, increased the release of this inflammatory cytokine into the culture medium. This indicates that factors upstream of testosterone, rather than testosterone itself, may mediate the activation of inflammation in these brain immune cells.

Our transcriptome analysis further confirmed that GABARs were the most dysregulated genes in the microglia, indicating that GABAA could be dysregulated in the neurons. GABAA is a well-known ion channel complex with anti-anxiety roles (Liu et al., 2018). In fact, the most widely used anti-anxiety drugs target the GABARs. In our previous study, we showed that progesterone regulated GABAR expression in sperm (Xu et al., 2017). Our current results further showed GABARs as the major targets of CYP11A1. This is the first study to demonstrate that CYP11A1 could play a key role in the regulation of GABAergic signaling and autism-like behavior. However, the exact mechanism through which CYP11A1 regulates GABAR expression requires further study. Additionally, studies are needed to clarify whether the altered expression levels of inflammation-related genes and GABAA are the outcomes of an altered hormone profile or inflammation-related genes in microglia that could contribute to changes in GABAA signaling in neurons. Furthermore, our previous study showed that progesterone could activate GABAA-delta through non-genomic means via interactions with P2X_2_ receptors (Xu et al., 2017).

In conclusion, our previous study had shown that CYP11A1 overexpression can induce preeclampsia and compromise placental development in rats. This study further provides potential insights into the mechanism through which CYP11A1 mediates autism-related neurobehavior and identifies key signaling targets for future intervention in the pathogenesis of autism related to abnormal hormone secretion during pregnancy.

## Data Availability Statement

The datasets presented in this study can be found in online repositories. The names of the repository/repositories and accession number(s) can be found below: https://www.ncbi.nlm.nih.gov/geo; GSE185805.

## Ethics Statement

The animal study was reviewed and approved by the Ethics Committee of Second University Hospital of Sichuan University, China.

## Author Contributions

XL, WX, and GH contributed to conception and design of the study. CJ and JC did the cell experiment parts and organized the database. JX performed the statistical analysis. TP performed the animal model and wrote the first draft of the manuscript. CJ and WX wrote sections of the manuscript. All authors contributed to manuscript revision, read, and approved the submitted version.

## Conflict of Interest

The authors declare that the research was conducted in the absence of any commercial or financial relationships that could be construed as a potential conflict of interest.

## Publisher’s Note

All claims expressed in this article are solely those of the authors and do not necessarily represent those of their affiliated organizations, or those of the publisher, the editors and the reviewers. Any product that may be evaluated in this article, or claim that may be made by its manufacturer, is not guaranteed or endorsed by the publisher.
